# Prospective Studies of Children of Alcoholic Parents

**Published:** 1997

**Authors:** Wendy Reich

**Affiliations:** Wendy Reich, Ph.D., is a research associate professor in the Department of Psychiatry, Washington University School of Medicine, St. Louis, Missouri

Alcoholism tends to run in families: Compared with children of nonalcoholics (non-COA’s), children of alcoholic parents (COA’s) have an approximately four times greater risk of becoming alcoholic themselves (West and Prinz 1987; Cotton 1979; Merikangas et al. 1985). The causal factors underlying the development (i.e., the etiology) of alcohol abuse and dependence, however, have not yet been conclusively determined. Studies conducted in the 1950’s and 1960’s generally emphasized psychosocial explanations, such as poor parenting, lack of good role models, and impoverished home life. Research performed since the 1970’s, however, also has investigated heritable components in the familial transmission of alcoholism. Adoption studies (Goodwin et al. 1973; Cadoret et al. 1985, 1987; Cloninger et al. 1981), analyses of half-siblings (Schuckit et al. 1972), and studies comparing identical (i.e., monozygotic) and fraternal (i.e., dizygotic) twins (Heath and Martin 1988; Heath et al. 1991; Kendler et al. 1992; McGue et al. 1992) have all provided evidence that genetic factors play a crucial role in the etiology of alcoholism.

Despite this strong evidence for a genetic contribution, few researchers would deny the influence of environmental factors in the development of alcoholism. The term “environmental” refers here to all factors that do not directly contribute to the genetic risk for alcoholism, whether they act in the prenatal period, early childhood, or early or late adolescence. Examples of such variables include maternal drinking during pregnancy, temperament and personality traits of the parents and children, psychopathology in the parents and children, geographic location, family and community environment, religious involvement, academic failure, and association with deviant peers. Because some of these factors (e.g., psychopathology) also have a genetic component, they may indirectly increase the genetic predisposition for alcoholism in some COA’s.

A major task in determining the etiology of alcoholism is to closely examine the interaction among the various environmental and genetic factors that determine a child’s pathway toward or away from alcoholism. Most likely, numerous such pathways exist, although only a limited number of critical variables may affect most of them (Sher 1994). The identification of these variables may help researchers distinguish “at-risk” populations that should be targeted for research and interventions aimed at prevention. To determine critical risk factors for alcoholism, particularly among COA’s, scientists have employed several study designs, including retrospective, cross-sectional, and prospective studies.

## Retrospective and Cross-Sectional Studies

Most of the information on the developmental pathways leading to alcoholism comes from retrospective or cross-sectional studies. Retrospective studies gather pertinent information about the subjects’ past based primarily on self-reports by the study participants. This information includes, for example, the age at which the subjects began to drink, the level and frequency of alcohol consumption, and the presence of certain types of psychopathology (e.g., depression and antisocial personality disorder [ASPD]). Although retrospective studies have yielded important information, the interpretation of these data often is limited because the subjects’ recall may not always be accurate. For example, although most study participants are able to report on their youth or young adulthood, they may forget certain events that occurred in the past or mistake the order in which the events occurred (e.g., whether the onset of depression preceded or followed the onset of alcohol problems). At least in some alcoholic subjects, these recall problems may be caused or aggravated by memory deficits resulting from long-term alcohol abuse.

Cross-sectional studies assess the subjects at one point in time. This method is useful in adults to determine traits that appear to be linked to alcoholism. Researchers also frequently use cross-sectional analyses to compare children or adolescents with alcoholic and nonalcoholic parents with respect to psychopathology, psychosocial stressors, and other environmental influences. In contrast to retrospective studies, cross-sectional analyses allow a much clearer picture of childhood precursors of alcoholism, because they assess relevant factors at the time they are happening. Furthermore, many of the instruments used in these studies also include questions about past experiences. Accordingly, cross-sectional studies can provide some of the same information as retrospective studies. Cross-sectional analyses, however, also have several limitations. For example, these studies cannot determine how environmental factors contributing to the risk for alcoholism (e.g., certain forms of psychopathology) and indicators of genetic risk (e.g., the number of alcoholics in the COA’s pedigree) interact to determine the COA’s outcome. In addition, people’s lives change over time, and COA’s whose parents stop drinking may experience different outcomes from those whose parents continue to drink. Consequently, the best way to study the pathways leading into alcoholism is to perform prospective studies that follow their subjects over extended periods of time.

## Prospective Studies of COA’s

Prospective studies record events at the time they are happening and then evaluate the results at a later time. For example, to determine how various environmental and genetic risk factors unfold and interact over time, prospective studies enroll COA’s early in life and follow them periodically until they are past or well into the age at which their likelihood of developing alcoholism is greatest (i.e., between the late teens and mid-thirties^1^). This approach generally is considered the most accurate, because it does not rely on the subjects’ recall of their past experiences and therefore involves the least guesswork. Moreover, the periodic sampling in prospective studies enables researchers to formulate specific questions throughout the study that can be answered in subsequent study phases. Finally, prospective studies generate descriptive data that help scientists and clinicians understand the natural history of alcoholism and other alcohol-related problems (Schuckit 1982).

Prospective studies nevertheless have several drawbacks. For example, they are time consuming and expensive. Furthermore, each study can assess only a limited number of factors without making the testing process too long and burdensome for the respondents and exceedingly expensive for the investigators. Before initiating a large-scale prospective investigation, researchers must therefore identify the most promising variables to be included, based on retrospective, cross-sectional, and small-scale prospective studies.

One classic prospective study of COA’s has investigated the hypothesis that people with close alcoholic relatives—particularly sons of male alcoholics—are more vulnerable to the effects of alcohol or respond differently to alcohol than do people without alcoholic relatives (Schuckit 1982, 1994; Schuckit and Smith 1996). This hypothesis was based on previous reports that the same amount of alcohol appeared to induce less intoxication in many sons of alcoholic fathers than in sons of nonalcoholic fathers. The first stage of this study included 304 male university students and nonfaculty employees who had responded to a questionnaire. Of these subjects, 44 (i.e., 14 percent) had at least one alcoholic first-degree relative (i.e., a parent or sibling) and were therefore designated as family history positive (FHP); the remaining subjects were classified as family history negative (FHN).

The FHP and FHN subjects did not differ in their drinking patterns: Both groups had similar proportions of drinkers (i.e., 95 percent and 89 percent, respectively), numbers of drinking days per week (i.e., 5.7 days and 6.2 days, respectively), and levels of alcohol consumption (i.e., 3.2 drinks per drinking day in both groups). Despite the similar levels of alcohol consumption, however, the FHP subjects experienced significantly more alcohol-related problems than did the FHN subjects, including academic problems, rejection by friends, automobile crashes, blackouts, unsuccessful attempts to stop drinking, problems with other drugs, and more serious behavior problems.

Based on these findings, the researchers decided to expand the study and include a prospective component. A second group of male college students responding to a questionnaire again were classified as FHP or FHN. In the prospective component in this study, the FHP subjects included only sons of alcoholic fathers. The investigators interviewed the study participants and administered a specific amount of alcohol to assess the subjects’ response to that dose (i.e., the level of intoxication). For each of the indicators of intoxication tested (e.g., performance in a motor task, levels of certain hormones, and electrophysiological changes in response to alcohol), FHP subjects overall exhibited less intense responses to alcohol compared with FHN subjects. Approximately 10 years later, the researchers conducted a followup to determine which of the subjects had developed alcoholism. Among both the FHP and FHN groups, subjects who had demonstrated a low intoxication level were more likely to have become alcoholic than were subjects who had exhibited greater intoxication in response to the same alcohol dose. Moreover, for each of the intoxication indicators previously tested, low scores predicted later alcohol problems.

This study provides an excellent example of the usefulness of the prospective design. If the investigators had solely conducted the first part of the study in a cross-sectional fashion, they could only have speculated about the likelihood that FHP subjects would develop alcoholism. Had the study only included the followup phase, the drinking histories of the alcoholic subjects might have made it difficult to determine the subjects’ initial level of response to alcohol. Only the prospective study design allowed the researchers to determine the correlation between sensitivity to alcohol’s intoxicating effects and the risk for alcoholism, thereby providing them with a tool to identify people who are at increased risk.

## Other Areas of Research for Prospective Studies of COA’s

A low-level response to alcohol is one biological marker identified in prospective studies as a likely indicator of a high risk for alcoholism. Other studies investigating differences between COA’s and non-COA’s to identify additional markers have focused on electrophysiological characteristics, such as event-related potentials (ERP’s). ERP’s are brain waves that can be measured during various cognitive tasks (e.g., recognizing a green light among a series of flashing red lights). Begleiter and colleagues (1984) have found that a certain component of the ERP wave (i.e., the P300 wave) is reduced in male COA’s compared with male non-COA’s. These data, which have been replicated many times, indicate a clear distinction between COA’s and non-COA’s. Prospective studies also have indicated that a reduced P300 wave is a good predictor of alcoholism.

Another critical area of research investigates the possibility that early childhood psychopathology might predict adult alcoholism. Most studies have demonstrated significantly higher levels of psychopathology—most commonly behavior disorders—in COA’s compared with non-COA’s. Together with findings that adult alcoholics frequently behave irresponsibly and that ASPD is the most common form of psychopathology in adult alcoholics, these observations suggest that in alcoholic families a certain continuity may exist between childhood and adulthood psychopathology. Although researchers have identified a potential relationship between childhood behavior disorders and adult alcoholism, they do not yet know which behavior disorders or combinations of disorders actually lead to alcoholism. Accordingly, one cannot predict which COA’s are at the highest risk or how childhood psychopathology interacts with the environment to moderate this risk. Consequently, prospective studies are needed that track and periodically interview COA’s and non-COA’s (both with and without psychopathology) and their families until the children are past the age at which they are most likely to develop alcoholism.

One ongoing prospective study that can address these issues is the Collaborative Study on the Genetics of Alcoholism (COGA). The main purpose of this study, which involves six research centers, is to search for genes that predispose people to different forms of alcohol abuse and dependence. The study’s subjects include alcoholics recruited at local treatment centers and as many of their relatives as possible, including children ages 7 and older. The investigators interview each subject, determine various biological and electrophysiological markers (e.g., ERP components), assess sensitivity to alcohol, and isolate DNA from each participant. The assessment of the children also includes an evaluation of any psychopathology, based on interviews with the child and a parent as well as on teacher reports. These analyses also provide information about the prenatal, perinatal, and preschool years. One goal of the study is to follow up with the subjects every 5 years. The study currently is in its first followup stage, in which researchers are reinterviewing some of the subjects enrolled 5 years ago. By monitoring the progress of the subjects, especially the children, over longer periods, the study should provide important information regarding the developmental pathways leading to alcoholism. If genes contributing to the development of alcoholism are identified, the study also can assess the COAs’ risk from a biological perspective by comparing their DNA with that of their parents. Ultimately, these data may help researchers formulate effective prevention measures for this devastating disorder.

## Footnotes

1 The recent literature indicates that the age of onset of alcoholism is generally getting younger.
